# Impact of animated instruction on tablets and hands-on training in applying bimanual perineal support on episiotomy rates: an intervention study

**DOI:** 10.1007/s00192-018-3711-6

**Published:** 2018-07-14

**Authors:** Kaled Mikki Zimmo, Katariina Laine, Erik Fosse, Mohammed Zimmo, Hadil Ali-Masri, Bettina Böttcher, Manuela Zucknick, Åse Vikanes, Sahar Hassan

**Affiliations:** 1 Department of Obstetrics, Al Aqsa Hospital, Gaza, Palestine; 2 The Intervention Centre, Oslo University Hospital, Rikshospitalet, 4950 Nydalen, 0424 Oslo, Norway; 3 Institute of Clinical Medicine, Faculty of Medicine, University of Oslo, Oslo, Norway; 4 Department of Health Management and Health Economics, Institute for Health and Society, University of Oslo, Oslo, Norway; 5 Department of Obstetrics, Oslo University Hospital, Ullevål, Oslo, Norway; 6 Department of Obstetrics, Al Shifa Hospital, Gaza, Palestine; 7 Department of Obstetrics, Palestine Medical Complex, Ramallah, Palestine; 8 Faculty of Medicine, Islamic University of Gaza, Gaza, Palestine; 9 Department of Biostatistics, Oslo Centre for Biostatistics and Epidemiology, University of Oslo, Oslo, Norway; 10 Departement of Nursing, Faculty of Pharmacy, Nursing and Health Professions, Birzeit University, Birzeit, Palestine

**Keywords:** Animated instructions video, Bimanual perineal support technique, Episiotomy rates, Hands-on, Palestine

## Abstract

**Introduction and hypothesis:**

In Palestine, episiotomy is frequently used among primiparous women.This study assesses the effect of training birth attendants in applying bimanual perineal support during delivery by either animated instruction on tablets or hands-on training on episiotomy rates among primiparous women.

**Methods:**

An interventional cohort study was performed from 15 October 2015 to 31 January 2017, including all primiparous women with singletons and noninstrumental vaginal deliveries at six Palestinian hospitals. Intervention 1 (animated instructions on tablets) was conducted in Hospitals 1, 2, 3, and 4. Intervention 2 (bedside hands-on training) was applied in Hospitals 1 and 2 only. Hospitals 5 and 6 did not receive interventions. Differences in episiotomy rates in intervention and nonintervention hospitals were assessed before and after the interventions and presented as *p* values using chi-square test, and odds ratios (OR) with 95% confidence intervals (CI). Differences in the demographic and obstetric characteristics were presented as *p* values using the Kruskal–Wallis test.

**Results:**

Of 46,709 women, 12,841 were included. The overall episiotomy rate in the intervention hospitals did not change significantly after intervention 1, from 63.1 to 62.1% (OR = 0.96, 95% CI 0.84–1.08), but did so after intervention 2, from 61.1 to 38.1% (OR = 0.39, 95% CI 0.33–0.47). Rates after Intervention 2 changed from 65.0 to 47.3% (OR = 0.52, 95% CI 0.40–0.67) in Hospital 1 and from 39.4 to 25.1% (OR = 0.49, 95% CI 0.35–0.68) in Hospital 2.

**Conclusions:**

Hands-on training of bimanual perineal support during delivery of primiparous women was significantly more effective in reducing episiotomy rates than animated instruction videos alone.

**Electronic supplementary material:**

The online version of this article (10.1007/s00192-018-3711-6) contains supplementary material, which is available to authorized users.

## Introduction

Episiotomy is a surgical procedure introduced to Europe in 1742 with the primary aim of enlarging the vaginal orifice to prevent severe perineal lacerations during childbirth [1]. Many recent studies confirm that restrictive use of episiotomy does not increase the risk of obstetric anal sphincter injuries (OASIS), and routine use is not recommended [2, 3]. The rate of episiotomy among primiparous women is described to vary between countries [4], ranging from 22.7% in Norway [5], up to 100% in Guatemala [4]. Previously described rates in Arab countries were 51.2% in Saudi Arabia [6], 66% in Oman [7], and 91% in Jordan [8]. Furthermore, a study from Palestine reported that routine episiotomy is practiced in six out of eight government hospitals during delivery of primiparous women [9]. Different perineal support techniques have been described in several studies, demonstrating positive impacts on reducing perineal trauma, although to a varying degree [5, 10, 11]. In a Norwegian study, the implementation of bimanual perineal protection reduced the use of episiotomy in spontaneous deliveries of primiparous women [5]. The bimanual perineal support technique (bPST) is a modified form of perineal support that slows down delivery of the fetal head at crowning with simultaneous protection of the posterior part of the perineum [5, 10, 11].

The impact of training by using mobile media, such as animated instructions on tablets, could be a possible way to overcome the outreach gap in areas where it is difficult to provide bedside training for health personnel in the best practice of medicine [12]. A recent survey among 124 physicians and midwives concluded that animation based on a bPST training video could be effective when applied in clinical practice [13]. Moreover, some studies compared hands-on training and use of mobile data and reported that training by mobile data as an educational tool showed the same effect when compared with hands-on training [12, 14]. Furthermore, the structured incorporation of electronic training, such as animated video, with hands-on training (face to face), has been shown to be an effective training approach in different medical fields [15].

Few previous studies described the effects of educational interventions on reduction of episiotomy rates [16, 17]. One study in a Palestinian maternity unit focused on the use of on-the-job training of healthcare providers and achieved a reduction of episiotomy rates from 80.0 to 39.1% [16]. The study presented here aimed to assess the effect of training birth attendants in the application of bimanual perineal support using animated instruction on tablets and hands-on training on the episiotomy rate among primiparous women at six hospitals in Palestine.

## Materials and methods

### Study design and settings

This was an interventional cohort study performed between 15 October 2015 and 31 January 2017 at six government hospitals in Palestine as part of the Palestinian Perineum and Birth Complications Study (PPS). The PPS was performed between March 2015 and April 2017 and included all women admitted for vaginal birth in six governmental hospitals in Palestine. The six hospitals—three in Gaza, Hospitals 1, 2, and 3; three in the West Bank, Hospitals 4, 5, and 6—were selected according to geographical distribution and the number of deliveries in each hospital [18]. Midwives and nurses work in the labor and delivery wards in Gaza hospitals. The characteristics for each hospital are shown in Table 1. Data from the PPS included information about mothers and children for vaginal deliveries and emergency and elective cesarean sections [18]. All data were collected from the District Health information system 2 (DHIS2) online software systems (http://www.pps.dhis2.org) and subsequently transferred to Services for Sensitive Data (TSD). TSD is a specific, secure platform developed by Oslo University for their researchers for collection, storage, analysis, and sharing of sensitive data to maintain security and privacy (tsd-drift@usit.uio.no).

**Table 1:** Characteristics of study hospitals^a^

	Hospital 1	Hospital 2	Hospital 3	Hospital 4	Hospital 5	Hospital 6
No. annual births	7500	4790	5800	6166	6798	12,500
No. maternity beds	34	32	20	43	40	80
No. ob/gyn doctors	16	5	5	3	8	35
No. residents	10	12	8	17	7	42
No. midwives	20	13	12	28	21	34
No. nurses	16	0	10	0	0	26

^a^Data from [18]

The study design, undertaking, and reporting followed the Standards for Quality Improvement Reporting Excellence (SQUIRE). The study was approved by the Palestinian Health Research Council (Reference no.: BHRC\HC\13\15), Regional Committee for Medical and Health Research Ethics in southeastern Norway (REK 2014/1727), and Norwegian Data Inspectorate (17/00082–2/GRA).

### Data collection

**Fig. 1:**
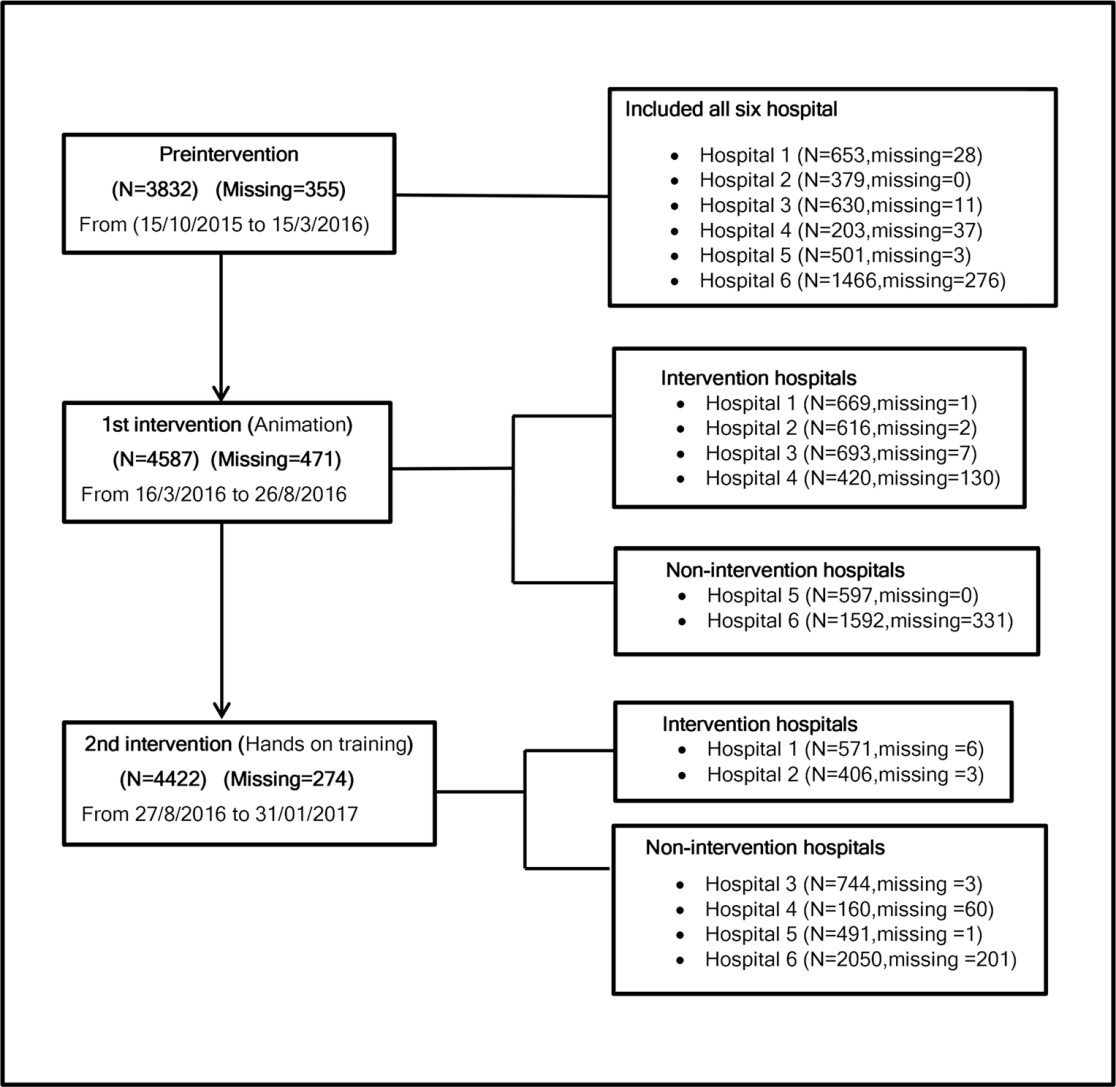
Study periods and hospital distributions

Data were collected prospectively by birth attendants (doctors and midwives). Each woman was registered on a specific case registration form (CRF) [18]. All completed forms were double-checked for availability and missing information using the hospitals’ formal records. All primiparous women (*n* = 12,841) with singleton noninstrumental vaginal deliveries (≥24 gestational weeks) between 15 October 2015 and 31 January 2017 were included in this study. Distribution of the population in each period of this study is presented in Fig. 1.

### Study phases

1. Phase one (Preintervention): 15 October 2015 to 14 March 2016
  This was an observational phase without any interventions, and the cumulative episiotomy rates were tracked in all study hospitals. During this phase, bPST was not introduced and not applied. However, some birth attendants regularly used one-handed perineal support techniques, mainly in instrumental deliveries. Furthermore, in Palestine, most women do not receive anesthesia during vaginal delivery, except for the local anesthesia (lidocaine) before an episiotomy cut. Pethidine may be used in some cases, which varies from one doctor to another but is usually not available in government hospitals. Mediolateral episiotomy is the common type used in the study hospitals.
2. Phase two (Intervention 1): 15 March 2016 to 27 August 2016
  This phase included the first intervention. One animated instruction video on tablets was used to demonstrate how to apply the bPST and to decrease the routine use of episiotomy. This intervention included four intervention hospitals (1, 2, 3, and 4) and two nonintervention hospitals (5 and 6). The video was designed by experts in this field specifically for this study with explanations in both Arabic and English (videos S1, S2). They were stored and presented on tablets. Two tablets for each intervention hospital were fixed in the labor wards and physicians’ meeting rooms to allow medical staff to watch the videos without interruption. Birth attendants were encouraged to watch the video as many times as they wanted, but no assistance or explanations were provided.
3. Phase three (Intervention 2): 28 August 2016 to 31 January 2017
  Hands-on training sessions were conducted by KL and two Norwegian midwives from Oslo University Hospital at two intervention hospitals (1 and 2). Hospitals 3, 4, 5, and 6 were the nonintervention hospitals in this phase. Almost 92% (84/92) of birth attendants in both intervention hospitals participated in the hands-on training and were taught the bPST on pelvic silicon models (face to face) after receiving lectures that illustrated the bimanual technique and emphasized decreasing the overuse of episiotomy. This part of the training was extended for 3 days in each intervention hospital to allow training as many birth attendants as possible, where those who were working on the first day were able to attend on the other days. This was followed by clinical supervision on the labor ward, where the instructor midwife guided the birth attendants in applying the support technique correctly. Then the birth attendants were asked to apply this technique themselves with complete supervision by the instructor midwife. One instructor midwife stayed in each hospital participating in Intervention 2 for 12 days, maximizing the opportunity for clinical training. However, no data is available on how many deliveries were done by each birth assistant applying the bPST under supervision of the instructor midwives or independently.

The bPST aims at controlling the speed of crowning by applying pressure on the fetal occiput with one hand. At the same time, the thumb and index finger of the other hand are used to support the perineum while the flexed middle finger makes a grip on the baby’s chin. As soon as a suitable grip is obtained, the woman stops pushing and breathes rapidly, while the birth attendant helps deliver the fetal head slowly through the vaginal introitus. When most of the head is delivered, the perineal ring is pushed under the baby’s chin (videos S1, S2).

### Statistical analysis

Data were analyzed using IBM SPSS (version 22, Chicago, IL, USA). Continuous variables are presented as means and standard deviation (SD). Differences between demographic and obstetric characteristics among the study population in each hospital were compared using *p* values of the Kruskal–Wallis test (Table 1). Categorical variables were presented as numbers and percentages in each phase of the study; *p* values of nonparametric chi-square tests assessed differences between hospitals among these variables. Differences between each phase of the study were evaluated using the *p* value of the chi-square test, odds ratio (OR), and 95% confidence intervals (CI). Statistical significance was defined as (two-sided) *p* values <0.05.

## Results

From a total of 46,709 women who gave birth during the study period, 13,852 (29.1%) were primiparous with singletons scheduled for vaginal delivery. All cases with instrumental vaginal deliveries were excluded: vacuum (*n* = 730) and forceps (*n* = 11). The study population consisted of all primiparous women with noninstrumental vaginal delivery (*n* = 12,841). Characteristics of the study population, such as maternal age, gestational age, and birth weight, were significantly different between hospitals. Small but consistent differences—in particular, for Hospitals 2 and 3—were observed in mean maternal age being several months higher in Hospital 2 for all intervention periods. At the same time, Hospitals 2 and 3 had women with clearly shorter gestational ages and slightly lower birth weights (together with Hospital 6). (Table 2).

**Table 2:** Characteristics of the study population across hospitals (*n* = 12,841) before, during ,and after interventions

	Hospital 1	Hospital 2	Hospital 3	Hospital 4	Hospital 5	Hospital 6	*P* value*
Study population per hospital							
Preintervention (*n* = 3832)	653	379	630	203	501	1466	< 0.001
Intervention 1 (*n* = 4587)	669	616	693	420	597	1592	< 0.001
Intervention 2 (*n* = 4422)	571	406	744	160	491	2050	< 0.001
Maternal age							
Preintervention	20.6 ± 4.1	23.4 ± 4.5	21.8 ± 3.9	22.6 ± 3.5	22 ± 4.3	22.1 ± 3.6	< 0.001
Intervention 1	22.5 ± 4.1	22.8 ± 4.0	21.5 ± 3.7	22.4 ± 3.4	21.8 ± 3.8	22.5 ± 3.5	< 0.001
Intervention 2	22.0 ± 3.9	23.0 ± 4.2	21.7 ± 3.9	22.1 ± 3.5	22.3 ± 3.9	22.1 ± 3.6	< 0.001
Gestational age at birth							
Preintervention	39.1 ± 1.7	38.3 ± 2.8	38.5 ± 4.7	38.9 ± 1.8	38.8 ± 1.9	39 ± 1.7	< 0.001
Intervention 1	39.1 ± 1.4	38.4 ± 2.9	38.5 ± 3.9	38.9 ± 1.7	38.7 ± 1.9	39.5 ± 1.9	< 0.001
Intervention 2	39.0 ± 1.3	38.3 ± 2.4	38.4 ± 4.4	38.9 ± 1.9	39.0 ± 1.8	39.3 ± 1.9	< 0.001
Birth weight							
Preintervention	3234 ± 407	3015 ± 534	3116 ± 674	3102 ± 311	3195 ± 420	3008 ± 691	< 0.001
Intervention 1	3264 ± 419	3079 ± 593	3081 ± 570	3255 ± 366	3160 ± 394	3094 ± 676	< 0.001
Intervention 2	3262 ± 421	3069 ± 555	3060 ± 574	3198 ± 381	3210 ± 402	3088 ± 534	< 0.001

Data are presented as *n* or (mean ± standard deviation). Intervention 1 conducted in Hospitals 1, 2, 3, and 4; Intervention 2 conducted in Hospitals 1 and 2

*P value, Kruskal–Wallis test or chi-square test

Following Intervention 1 (video animation), no statistical difference was found in episiotomy rates in intervention hospitals when compared with the preintervention period (from 63.1 to 62.1%, OR = 0.96, 95% CI 0.84–1.08) and in nonintervention hospitals (Hospitals 5 and 6) during this phase (from 79.6 to 80.5%, OR = 1.06, 95% CI 0.91–1.23). In intervention hospitals, a significant decrease in episiotomy rate was found in Hospital 1, from 70.8 to 65% (OR = 0.77, 95% CI 0.61–0.97) compared with a nonsignificant decrease in Hospital 2, from 44.6 to 39.4% (OR = 0.81, 95% CI 0.63–1.04). On the other hand, the episiotomy rate significantly increased in Hospital 3, from 61.7 to 69.3%, and showed no change in Hospital 4 after Intervention 1. No changes were found in Hospital 5 and 6, where no intervention was applied. (Table 3).

**Table 3:** Episiotomy rates across hospitals before and after Intervention 1

Hospitals	Preintervention	Intervention 1^a^	OR	95% CI		*P* value*
	*N*/Total (%)	*N*/Total (%)		Lower	Upper	
Intervention hospitals	1176 /1865 (63.1)	1488/2398 (62.1)	0.96	0.84	1.08	0.50
Hospital 1	462/653 (70.8)	435/669 (65.0)	0.77	0. 61	0.97	0.03
Hospital 2	169/379 (44.6)	243/616 (39.4)	0.81	0.63	1.04	0.11
Hospital 3	389/630 (61.7)	480/693 (69.3)	1.39	1.11	1.75	0.004
Hospital 4	156/203 (76.8)	330/420 (78.6)	1.10	0.74	1.64	0.63
Nonintervention hospitals	1566/1867 (79.6)	1763/2189 (80.5)	1.06	0.91	1.23	0.46
Hospital 5	333/501 (66.5)	406/597 (68.0)	1.07	0.83	1.38	0.59
Hospital 6	1233/1466 (84.1)	1357/1592 (85.2)	1.09	0.89	1.32	0.38

^a^Intervention 1 conducted in Hospitals 1, 2, 3, and 4

*P value of chi-square test

There was a discrepancy between hospitals conducting Intervention 1 in the number of times the animated instruction video was watched by birth attendants. In Hospitals 1 and 3, the team had watched the animation more frequently than in Hospitals 2 and 4 (Supplementary Fig. 1). After Intervention 2 (hands-on training), the episiotomy rate in intervention hospitals (1 and 2) was significantly reduced when compared with the preintervention period, from 61.1 to 38.1% (OR = 0.39, 95% CI 0.33–0.47). Specifically, the observed reduction in Hospital 1 was from 70.8 to 47.3% (OR = 0.37, 95% CI 0.29–0.47) and in Hospital 2 from 44.6 to 25.1% (OR = 0.42, 95% CI 0.31–0.56). Moreover, no stastistically significant differnce was observed in episiotomy rates between nonintervention hospitals (3, 4, 5, and 6), from 75.4 to 77.9% (OR = 1.08, 95% CI 0.96–1.21) (Table 4).

**Table 4:** Episiotomy rates across hospitals before and after Intervention 2

Hospitals	Preintervention	Intervention 2^a^ (hands-on)	OR	95% CI		*P* value^b^
	*N* (%)	*N* (%)		Lower	Upper	
Intervention hospitals	631/1032 (61.1)	372/977 (38.1)	0.39	0.33	0.47	< 0.001
Hospital 1	462/653 (70.8)	270/571 (47.3)	0.37	0.29	0.47	< 0.001
Hospital 2	169/379 (44.6)	102/406 (25.1)	0.42	0.31	0.56	< 0.001
Nonintervention hospitals	2111/2800 (75.4)	2644/3445 (76.7)	1.08	0.96	1.21	0.21
Hospital 3	389/630 (61.7)	491/744 (66.0)	1.20	0.96	1.50	0.10
Hospital 4	156/203 (76.8)	136/160 (85.0)	1.70	0.99	2.94	0.06
Hospital 5	333/501 (66.5)	312/491 (63.5)	0.88	0.68	1.14	0.33
Hospital 6	1233/1466 (84.1)	1705/2050 (83.2)	0.93	0.78	1.12	0.46

^a^Intervention 2 conducted in Hospitals 1 and 2

*P value of chi-square test

Following Intervention 2, in comparison with Intervention 1, significant decreases in episiotomy rates were observed in Hospitals 1—from 65.0 to 47.3% (OR = 0.52, 95% CI 0.40–0.67) and 2—from 39.4 to 25.1% (OR = 0.49, 95% CI 0.35–0.68). However, no significant changes were observed in the remaining four (Hospitals 3, 4, 5, and 6, where Intervention 2 was not implemented (Table 5).

**Table 5:** Episiotomy rates across hospitals during Interventions 1 and 2

Hospitals	Intervention 1^a^	Intervention 2^b^	OR	95% CI		*P* value*
	N (%)	N (%)		lower	upper	
Hospital 1	435/669 (65.0)	270/571 (47.3)	0.52	0.40	0.67	< 0.001
Hospital 2	243/616 (39.4)	102/406 (25.1)	0.49	0.35	0.68	< 0.001
Hospital 3	480/693 (69.3)	491/744 (66.0)	0.86	0.67	1.10	0.19
Hospital 4	330/420 (78.6)	136/160 (85.0)	1.59	0.92	2.76	0.10
Hospital 5	406/597 (68.0)	312/491 (63.5)	0.77	0.58	1.03	0.12
Hospital 6	1357/1592 (85.2)	1705/2050 (83.2)	1.10	0.88	1.36	0.10

^a^Intervention 1 conducted in Hospitals 1, 2, 3 and 4

^b^Intervention 2 conducted in Hospitals 1 and 2

*P value of chi-square test

## Discussion

Hands-on and bedside training of bPST resulted in a significant reduction of episiotomy use, by almost 38%, among practitioners delivering primiparous women in the intervention hospitals compared with control hospitals. Furthermore, education by animated instructions on tablets had no observed impact on episiotomy rates in the intervention hospitals. Before the interventions, episiotomy rates among primiparous women varied across hospitals but were considered high in all hospitals, except for Hospital 2. A previous study had shown a significant reduction in the use of episiotomy among primiparous women, from 80 to 39.1% in this same hospital [16], reflecting results of successful long-term efforts to limit the overuse of episiotomy.

Mobile health-training tools, such as animated instruction videos, have become common for educational purposes. This provided new opportunities for developing skills and gaining knowledge in a clinical setting [19]. It allows trainees to watch and learn at any time, at any place, as many times as they want, even without the need for Internet connection. The latter is important for settings with poor Internet availability, such as Gaza [12, 13]. Moreover, a survey conducted among 1508 American residents and medical students reported that most medical students and trainees were in favor of mobile health-technology tools as being one of the leading components of their training program [20]. Another study, from the UK, reported that using tablets as a training tool was valuable for training as well as enhancing laparoscopic surgical skills, with reduced costs when compared with hands-on training [21]. In contrast, results of our study showed no effect of animated instructions presented on tablets on the reduction of episiotomy rates. Possible explanations may be that birth attendants did not follow the presented delivering technique, or it could be due to a dislike of the technique, or that they were unfamiliar with the use of this technology, or that they we not sure how to apply the technique without guidance and support.

The use of mixed interventions, such as interactive video, hands-on, and noninteractive video training as teaching methods in the management of postpartum hemorrhage, appeared to have similar effects, with a potential advantage of video training, which provides more available use, especially in remote areas [14]. In Palestine, as a country with scarce resources, video technology or animated instructions could offer a possible way to promote and implement evidence-based practice. However, findings of our study do not explicitly support an approach to changing educational practice into more use of animated instructions in Palestine. This could be due to the heavy workload, lack of time and motivation, as well as fear of responsibility for ensuing complications, such as OASIS, when implementing a new technique.

The role of hands-on perineal support and hands-off techniques during delivery are still under review [22]. The Royal College of Obstetricians and Gynecologists considered both hands-on perineal support or hands-off delivery techniques as appropriate methods for easing spontaneous vaginal deliveries, highlighting that perineal protection at crowning could be protective [23]. On the other hand, the American College of Obstetricians and Gynecologists’ Practice Bulletin did not recommend the routine use of manual perineal support during vaginal deliveries [24]. Based on results of reduced OASIS rates in Norway and Denmark after implementing manual perineal protection, which primarily focused on decreasing OASIS rates rather than episiotomy rates, the technique was implemented in Palestine [5, 11, 25–27]. Two Norwegian studies confirmed a significant decrease in OASIS rates in the study hospitals, while the episiotomy rate increased in some hospitals and remained unchanged in the others. These studies did not differentiate between instrumental and noninstrumental deliveries [11, 25]. However, in another Norwegian study that did so, results showed a reduction of episiotomy use in spontaneous deliveries when bPST was applied but increased in instrumental deliveries during the same period [5]. In the study we present here, we included women with singleton noninstrumental deliveries only.

Moreover, two Danish studies, also including instrumental and spontaneous vaginal deliveries, reported conflicting results. One study concluded that performing bPST during delivery reduced the OASIS incidence without increasing the episiotomy rates [27]. The other study reported an increase of episiotomy use from 4.4 to 7.1% when bimanual perineal support was reintroduced as routine practice [26]. Variations in the impact of bPST implementation on episiotomy rates could partly be explained by different baseline levels of episiotomy use in the Danish (<10%), the Norwegian (15–25%), and our study (70.2%). It is presumably easier to reduce episiotomy rates from high levels (70%) when compared with already low levels (<10% and 15–25% respectively)—meaning that a greater number of women need to be included in low-prevalence settings to show significant differences. By introducing additional hands-on training in the bPST, the episiotomy rate was reduced from 61.1 to 38.1% in our study.

The strengths of this study are the large population size of primiparous women and the comprehensive database including detailed information on all pregnancies and births scheduled for vaginal delivery, which reduced the risk of selection bias. Furthermore, most deliveries in Palestine take place in governmental hospitals, which makes the study findings representative and generalizable. Additionally, it is a prospective population-based study, reducing the risk of information bias.

Limitations of this study include missing data on some variables; the proportion of missing data varied across study hospitals. Since missing data were random, they are not believed to affect outcomes. A short time for follow-up of episiotomy rates after each intervention, of only 6 months, cannot test the long-term effect of the intervention. Additionally, other hospital-specific factors, such as obstetric practices and culture, could have affected data. Furthermore, exposure to the training video was not standardized, but varied among heathcare staff in the different hospitals. This variation in frequency of watching the training video, ranging from 58 to 255 (Fig. S1), could have negatively influenced the effectiveness of this intervention. A more structured approach in the use of animated instructions of the bPST with a defined minimum could have led to greater effectiveness of this intervention but was too difficult to implement in this study.

Additionally, Hospitals 1 and 2 are the workplaces for KZ and HAM, which could potentiate the outcome of interventions by applying continuous support and guidance for birth attendants during daily practice. In this study, two educational interventions were used for the same technique. Therefore, one intervention may have reinforced the effect of the other. It is challenging to verify the exact part educational interventions played in reducing episiotomy rates. However, many additional factors may have affected them, such as birth attendants’ experience, extent of patient response to the attendant’s guidance, and birth position [25]. However, this study showed that hands-on training of birth attendants in employing bPST is an effective tool for reducing episiotomy rates.

## Conclusions

Hands-on training of birth attendants in the bPST significantly reduced episiotomy rates in primiparous women and was considerably more effective than video animation training alone. To generalize the potential benefit of reduced episiotomy rates, hands-on training of birth attendants in bPST must be continuously implemented and reach more hospitals.

## Electronic supplementary material

ESM 1 The animated bimanual perineum support technique training video (English version). MP4; 19.8 MB. Duration: 5.40 min. The video is used with permission from Oslo University. (MP4 18,988 kb)

ESM 2 The animated bimanual perineum support technique training video (Arabic version). MP4; 19. 8 MB. Duration: 5.40 min. (MP4 12,538 kb)

ESM 3 (PDF 413 kb)

## Abbreviations

bPST: Bimanual perineal support technique
OASIS: Obstetrical anal sphincters injuries
