# Supplementary material for: Impact of animated instruction on tablets and hands-on training in applying bimanual perineal support on episiotomy rates: an intervention study
Source: Int Urogynecol J. 2018 Jul 14;30(8):1343–50. doi: 10.1007/s00192-018-3711-6 (PMC6647226; doi:10.1007/s00192-018-3711-6)
Supplement: Supplementary file 3 — (PDF 413 kb) [file 192_2018_3711_MOESM3_ESM.pdf]

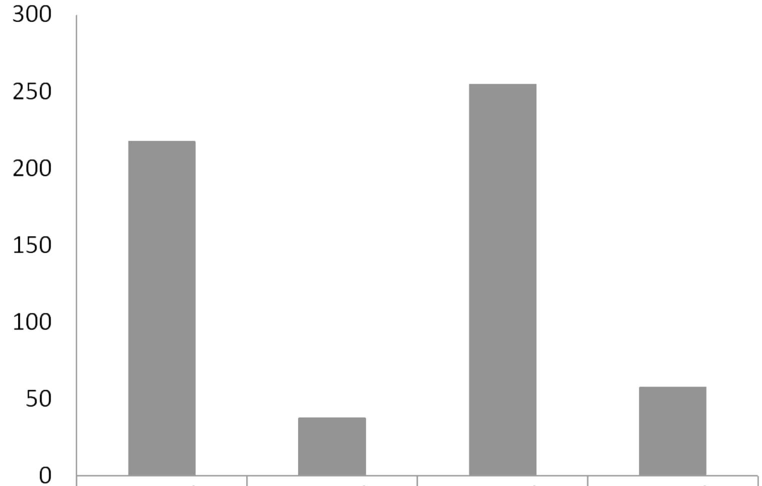


| | Hospital 1 | Hospital 2 | Hospital 3 | Hospital 4 |
|---|---|---|---|---|
| number of times | 218 | 38 | 255 | 58 |

**Figure S1: Number of times animated instruction video watched per hospitals (from 15th of March to 27th of August 2016)**
